# Technical-Economical Study on the Optimization of FDM Parameters for the Manufacture of PETG and ASA Parts

**DOI:** 10.3390/polym16162260

**Published:** 2024-08-09

**Authors:** Dragos Valentin Iacob, Dragos Gabriel Zisopol, Mihail Minescu

**Affiliations:** 1 Department of Mechanical Engineering, Doctoral School, Petroleum-Gas University of Ploiesti, 100680 Ploiesti, Romania; dragoshicb@gmail.com; 2 Mechanical Engineering Department, Petroleum-Gas University of Ploiesti, 100680 Ploiesti, Romania

**Keywords:** three-dimensional printing, FDM, value analysis, printing parameters, optimization, PETG, ASA, tensile, compressive

## Abstract

The article presents the results of the technical–economical study regarding the optimization of fused deposition modeling (FDM) parameters (the height of the layer deposited in one pass—L_h_ and the filling percentage—I_d_) for the manufacture of Polyethylene Terephthalate Glycol (PETG) and Acrylonitrile Styrene Acrylate (ASA) parts. To carry out this technical–economical study, was used the fundamental principle of value analysis, which consists of maximizing the ratio between *V_i_* and *C_p_*, where *V_i_* represents the mechanical characteristic, and *C_p_* represents the production cost. The results of the study show that for tensile specimens made of PETG, the parameter that significantly influences the results of the *V_i_*/*C_p_* ratios is the height of the layer deposited in one pass, (L_h_), and in the case of the compression specimens made of PETG, the parameter that significantly influences the results of the *V_i_*/*C_p_* ratios is filling percentage (I_d_). In the case of specimens manufactured via FDM from ASA, the parameter that decisively influences the results of the *V_i_*/*C_p_* ratios of the tensile and compression specimens is the filling percentage (I_d_). By performing optimization of the process parameters with multiple responses, we identified the optimal parameters for FDM manufacturing of parts from PETG and ASA: the height of the layer deposited in one pass, L_h_ = 0.20 mm, and the filling percentage, I_d_ = 100%.

## 1. Introduction

Additive manufacturing consists of making components by successively adding material layer by layer, according to the instructions specified by the G-Code file [1,2,3,4]. Additive manufacturing technologies have experienced continuous evolution since their inception, and due to their significant advantages over formative and subtractive technologies, these technologies are now widely used in many industrial sectors [5,6,7,8,9,10,11,12,13,14,15,16,17]. The major advantages of additive manufacturing technologies are represented by the efficiency of the use of materials (the amount of technological residues is negligible), the manufacturing of complex geometries without basing and fixing elements, and the low consumption of electricity [18,19,20,21,22,23,24,25,26,27,28].

The additive manufacturing process represents a major innovation in the field of manufacturing, enabling the transformation of digital concepts into physical objects [29,30,31,32,33,34]. This process encompasses several essential steps to ensure the transition from digital design to the final physical product:

- CAD conceptualization;
- Saving the CAD model and converting it into STL format;
- Generating the G-Code file;
- Equipment preparation, construction, extraction and use of parts.

In [4], innovative strategies are presented for the technical–economical optimization of the parameters of 3D printing via FDM (L_h_—the height of the deposited layer in one pass; I_d_—the filling percentage). To optimize the parameters, the value analysis method was used, which consists of maximizing the ratio between the use value (*V_i_*) and the production cost (*C_p_*). The use value is represented by the mechanical characteristics. The results of the study show that of the two parameters considered (L_h_ and I_d_), the height of the layer deposited at one pass decisively influences the bending resistance, and I_d_ categorically influences the resistance to breaking and compression, but also the hardness. The optimal parameters for printing PLA via FDM are L_h_ 0.15 mm and I_d_ 100%, those for heat-treated PLA are 0.20 mm and I_d_ 100%, and those for ABS are L_h_ 0.15 mm and I_d_ 100%.

In [35], the authors present a study on the optimization of FDM parameters (I_d_—filling percentage; L_h_—height of the deposited layer in one pass; W_n_—number of walls; E_t_—extruder temperature; P_s_—printing speed; B_t_—platform temperature; N_l_—the number of lower and upper layers; I_p_—the filling pattern) to minimize energy consumption, but without affecting the traction characteristics. The conclusions show that the parameters that categorically influence the energy consumption, but also the traction characteristics, are I_d_, L_h_, W_n_, and B_t_, and their optimal values are as follows: I_d_—90%; L_h_—0.30 mm; W_n_—4; B_t_—60 °C.

In [36], a study is presented on the optimization of FDM parameters (N_d_—extrusion nozzle diameter; W_n_—number of walls; E_t_—extruder temperature; I_d_—filling percentage; I_p_—filling pattern) to reduce the printing time, but without affecting the mechanical properties of the parts. The results of the study show that the parameters that decisively influence the printing time are as follows: N_d_, I_d_, and W_n_. Research suggests that a larger nozzle diameter (N_d_ = 0.60 mm), four outer shells (W_n_ = 4), and a 10% infill (I_d_ = 10%) can reduce print time without compromising mechanical characteristics.

In [37], the authors presents a study regarding the impact of FDM parameters (L_h_—height of the deposited layer in one pass; E_t_—extruder temperature; P_s_—printing speed; B_t_—platform temperature) on the compression behavior of samples made of PLA filament, and its use in biomedical and clinical applications is investigated. The considered FDM parameters significantly influenced the mechanical properties, and statistical simulations and SEM (scanning electron microscopy) analyses showed the ability to improve the mechanical properties. The conclusions of the study show that the highest value of the compressive strength (C_s_) was obtained for the samples made with the following parameters: L_h_ = 0.10 mm; T_e_ = 205 °C, B_t_ = 60 and P_s_ = 50 mm/min. ANOVA certified that the parameter that decisively influences the compressive strength (C_s_) is the height of the layer deposited at one pass (L_h_).

In [9], a study is presented on the influence of the filling pattern (I_p_) on the compressive strength of parts manufactured via FDM from PLA. In this context, 28 samples were manufactured on the Anycubic 4 Max Pro 2.0 3D printer using seven filling patterns (Grid, Tri-Hexagon, Octet, Triangles, Cubic subdivision, Gyroid, Cross-3D). The dimensions of the specimens were measured before and after the compression test using a DeMeet 3D coordinate measuring machine. The results show a minimum printing accuracy of 98.98% and a maximum deformation value of 57.70% for the specimens with the Triangles fill pattern. The highest values of compressive strengths were obtained for the specimens with the Triangles filling pattern. To establish the optimal option from a technical–economical point of view, a maximization of the ratio between the use value (*V_i_*) and the production cost (*C_p_*) was carried out, this ratio being one of the fundamental technical–economical principles of the value analysis. The Cubic subdivision fill pattern is the most efficient method for the FDM fabrication of PLA compression specimens using lattice structures.

Additive manufacturing technologies have significant potential to contribute to sustainability in various industries, with the main benefits being waste reduction, optimizing the use of materials, reducing energy consumption, the use of sustainable materials, and integration of the production and consumption model based on circularity [38,39,40,41,42,43,44,45,46,47,48,49,50,51].

Table 1 details the main additive manufacturing technologies, including the components, operating principles, and advantages and disadvantages of each technology.

In the following, attention will be paid to the use of additive manufacturing technology through thermoplastic extrusion, this being one of the most widespread additive manufacturing technologies due to its ease of use, but also because of its low costs of equipment and materials (see Table 1).

This paper presents a technical–economical study regarding the optimization of the FDM parameters (L_h_—the height of the layer deposited in one pass and I_d_—the filling percentage) for the manufacture of tensile and compression specimens from PETG and ASA. The novelty of this study consists in the application of the fundamental principle of value analysis (AV), which aims to maximize the ratio between the use value (*V_i_*) and the production cost (*C_p_*). Thus, we will establish the optimal FDM parameters for the manufacture of tensile and compression specimens from PETG and ASA.

## 2. Materials and Methods

The variable parameters of FDM used in the manufacture of tensile and compression specimens from PETG and ASA are the height of the deposited layer in one pass, L_h_ = (0.10/0.15/0.20) mm and the filling percentage I_d_ = (50/75/100)%. The mechanical properties of tensile (tensile strength, percentage elongation at break and elastic modulus) and compressive (compressive stress), were previously determined by the authors in works [48,49], respectively [50,51].

Using the parameters from Table 2, 54 tensile specimens (27 of PETG and 27 of ASA), in accordance with [52], and 90 compression specimens (45 of PETG and 45 of ASA) were manufactured on the Anycubic Pro Max 2.0 3D printer (Shenzhen, China), in accordance with [53]. Tensile and compression specimens made from Everfil PETG and ASA filament on the Anycubic Pro Max 2.0 3D printer were tested on the Barrus White 20 kN universal testing machine.

Table 3 shows the technical specifications of the Everfil filament used in the manufacture of tensile and compression specimens from PETG and ASA. Adapted with permission from [54,55], 2024, 3DKORDO.

Following the realization of the experimental determinations for the two types of mechanical tests (tension and compression), as well as the calculation of the production cost for each set of samples, a technical–economical study on the optimization of the FDM parameters was carried out. To establish the optimal variant, the fundamental principle of value analysis was used, which is presented in relation 1, and which consists of maximizing the ratio between the use value (*V_i_*) and the production cost (*C_p_*) [4,9,56,57,58,59]. This fundamental principle of value analysis is an effective tool for multi-objective optimization processes because it is value-driven and cost-effective, and it simplifies the decision process because it offers clear and easy-to-follow objective functions.

(1) $$\frac{V_{i}}{C_{p}}\to max$$

where *V_i_* represents the value in use (mechanical characteristic), and *C_p_* represents the production cost expressed in monetary units.

Minitab 19 software was used to optimize the ratio between *V_i_* and *C_p_*. To calculate the production cost, the following relationship was used [4,9,56,57,58,59]:

(2) $$C_{p}=Q_{mat}\times P_{mat}+P_{t}\times E_{c}\times P_{en}$$

where $C_{p}$ represents the production cost (EUR); $C_{mat}$ represents the cost of the material (EUR); $C_{en}$ represents the energy cost (EUR); $Q_{mat}$ represents the quantity of used material (g); $P_{mat}$ represents the material price (EUR/g); $P_{t}$ represents the printing time (h); $E_{c}$ represents the energy consumption (kW); $P_{en}$ represents the price of electrical energy (euro/kWh).

The following constant values were used to perform the economical calculations: $P_{m}$ = 0.22 Euro/g (for PETG); $P_{m}$ = 0.23 Euro/g (for ASA); $P_{en}$ = 0.25 kW/h; $E_{c}$ = 0.23 kW/h [60]. The material consumption and print time values for each set of samples were generated by Cura Slicer version 5.7.2, [61].

The dimensions and test conditions of the tensile and compression specimens are shown in Table 4.

## 3. Results and Discussion

### 3.1. Applications of Value Analysis for Analyzing the Mechanical Behavior of PETG and ASA 3D-Printed Samples

#### 3.1.1. Tensile Testing

Table 5 and Table 6 show the results obtained from the application of relation 2 and the determination of the production cost for the tensile specimens manufactured via FDM from PETG and ASA.

FDM parameters impact the mechanical behavior of parts made of PETG and ASA, but also the consumption of electricity [35]. The results of the *V_i_*/*C_p_* ratio are shown in Table 7 and Table 8.

**Figure 1:**
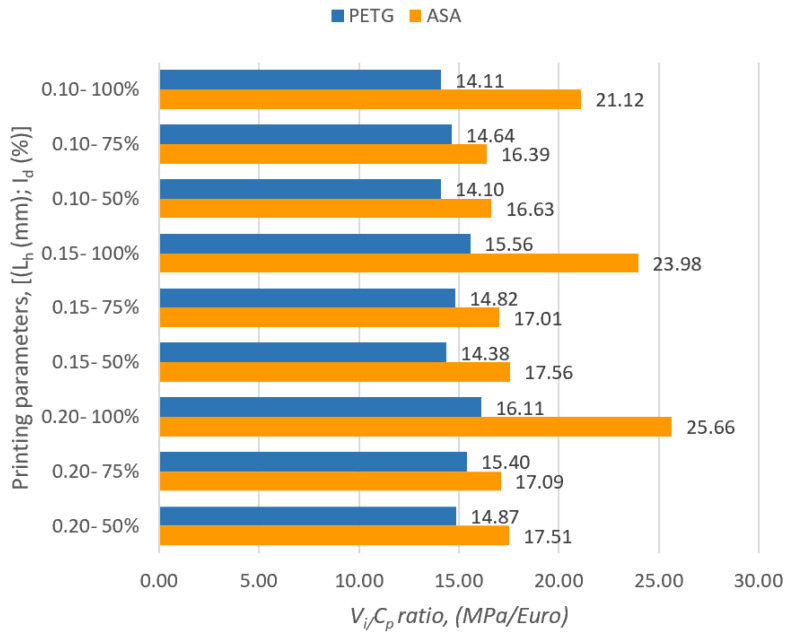
Determination of *V_i_*/*C_p_* ratio for tensile samples made from PETG and ASA.

Figure 1 graphically shows the values of the ratios between *V_i_* (ultimate tensile strength) and *C_p_* (production cost) of the samples manufactured via FDM from PETG and ASA.

Analyzing Figure 1, we notice that the highest value of the ratio between *V_i_* (ultimate tensile strength) and *C_p_* (production cost) was obtained for the set of specimens made of ASA with the layer height deposited at a pass of L_h_ = 0.20 mm and with a percentage of filling of I_d_ = 100%. In the case of specimens made of PETG, the highest value of the ratio between *V_i_* and *C_p_* was obtained for the set of specimens with the layer height deposited at a pass of L_h_ = 0.20 mm and with a filling percentage of I_d_ = 100%. Comparing the minimum and maximum results of the *V_i_*/*C_p_* ratios of the ASA samples with those obtained for the PETG samples, it was found that for the ASA samples, the results are 13.94–37.23 % higher compared to the results of the *V_i_*/*C_p_* ratios of the samples made from PETG.

**Figure 2:**
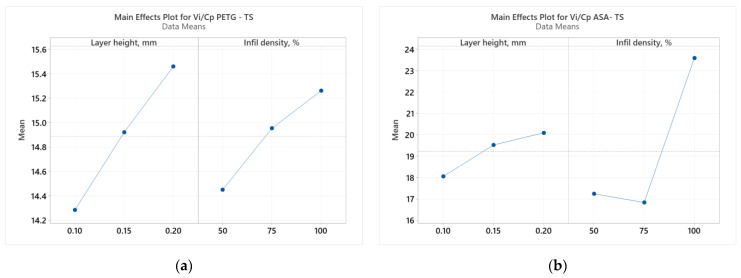
Main effects plots for tensile strength: (**a**) PETG; (**b**) ASA.

Using the Minitab 19 software, we performed ANOVA (analysis of variances), which includes sets of statistical methods and procedures used to analyze differences between means, [62]. In our study, we used ANOVA to evaluate the relationship between the FDM parameters (L_h_ and I_d_) and the result of the ratio between *V_i_* (ultimate tensile strength) and *C_p_* (production cost) [42]. Figure 2 shows the result of the ANOVA.

**Figure 3:**
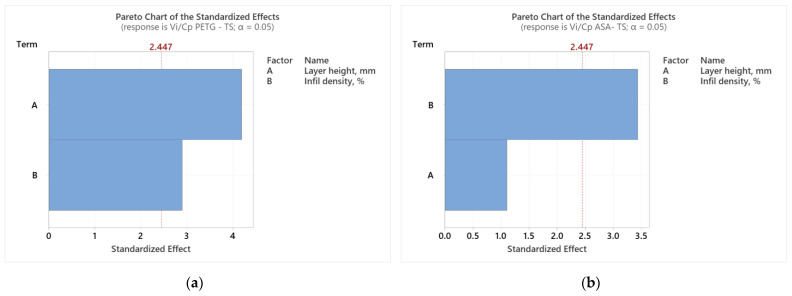
Pareto charts for tensile strength: (**a**) PETG; (**b**) ASA.

Analyzing Figure 2, we observe how the two considered parameters (L_h_ and I_d_) affect the result of the *V_i_*/*C_p_* ratio of the tensile specimens made of PETG (Figure 2a) and ASA (Figure 2b). According to Figure 2a, the layer height deposited in one pass (L_h_) was the parameter that significantly influenced the result of the *V_i_*/*C_p_* ratio of the tensile specimens made of PETG. Analyzing Figure 2b, we notice that the filling percentage (I_d_) was the parameter that decisively influenced the result of the *V_i_*/*C_p_* ratio of the tensile specimens made of ASA. The same conclusions are suggested by the Pareto charts shown in Figure 3.

#### 3.1.2. Compressive Testing

Table 9 and Table 10 present the results obtained following the application of relation 2 and the determination of the production cost for the compression specimens manufactured via FDM from PETG and ASA.

Table 11 and Table 12 show the *V_i_*/*C_p_* results for the compression specimens manufactured via FDM from PETG and ASA.

**Figure 4:**
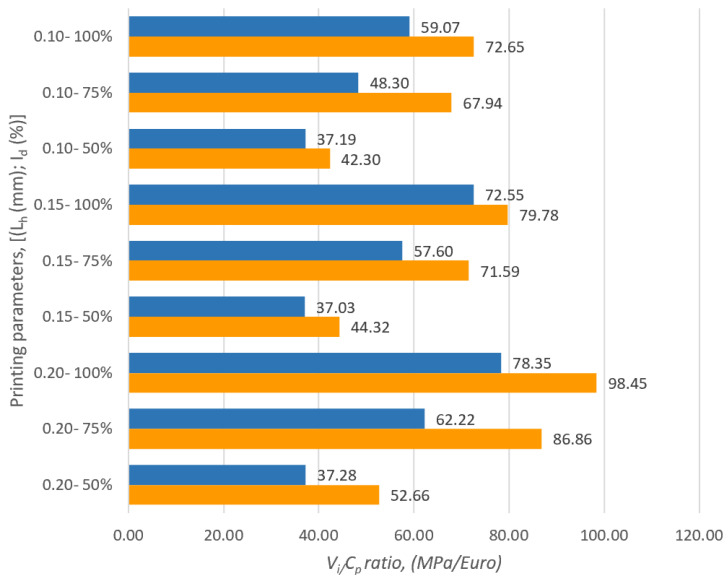
Determination of *V_i_*/*C_p_* ratio for compressive samples made from PETG and ASA.

Figure 4 graphically shows the values of the ratios between *V_i_* (compressive strength) and *C_p_* (cost of production) of the samples manufactured via FDM from PETG and ASA.

Analyzing Figure 4, we notice that the highest value of the ratio between *V_i_* (compressive strength) and *C_p_* (cost of production) was obtained for the set of samples made of ASA with the height of the layer deposited at a pass of L_h_ = 0.20 mm and with a filling percentage of *I_d_* = 100%. In the case of specimens made of PETG, the highest value of the ratio between *V_i_* and *C_p_* was obtained for the set of specimens with the layer height deposited at a pass of L_h_ = 0.20 mm and with a filling percentage of I_d_ = 100%. Comparing the minimum and maximum results of the *V_i_*/*C_p_* ratios of the ASA samples with those obtained for the PETG samples, it is found that for the ASA samples the results are 12.47–20.42% higher compared to the results of the *V_i_*/*C_p_* ratios of the samples made from PETG.

**Figure 5:**
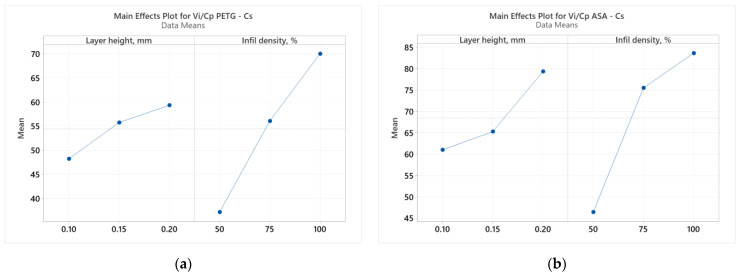
Main effects plots for compressive strength: (**a**) PETG; (**b**) ASA.

Figure 5 shows the results of the ANOVA, during which the relationship between the FDM parameters (L_h_ and I_d_) and the result of the ratio between *V_i_* (compressive strength) and *C_p_* (production cost) was studied.

**Figure 6:**
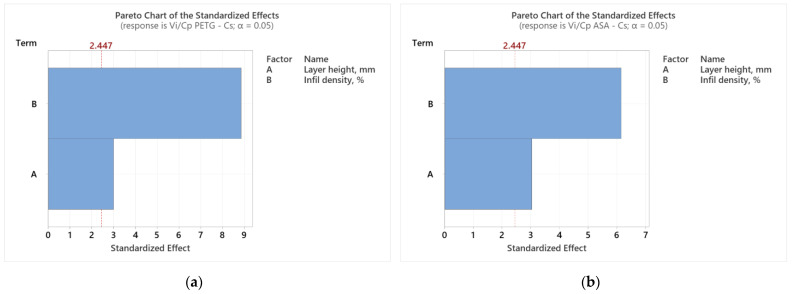
Pareto charts for compression strength: (**a**) PETG; (**b**) ASA.

Analyzing Figure 6, we observe how the two considered parameters of FDM (L_h_ and I_d_) affect the result of the *V_i_*/*C_p_* ratio of compression specimens made of PETG (Figure 5a) and ASA (Figure 5b). According to Figure 6a, the filling percentage (I_d_) is the parameter that significantly influences the *V_i_*/*C_p_* ratio result of compression specimens made of PETG. Analyzing Figure 5b, we notice that the filling percentage (I_d_) is the parameter that decisively influences the result of the *V_i_*/*C_p_* ratio of the ASA compression specimens. The same conclusions are suggested by the Pareto charts shown in Figure 6.

### 3.2. Optimization of FDM Parameters Based on Value Analysis for Improving the 3D Printing Efficiency for Samples Made of PETG and ASA

Using Minitab 19, the FDM parameters presented in Table 2 and the results obtained via applying the fundamental principle of value analysis by maximizing the *V_i_*/*C_p_* ratio, we optimized the FDM parameters with the aim of achieving technical–economical efficiency.

To optimize the FDM parameters, we used the desirability method, where the goal was to maximize the values of the ratios between *V_i_* and *C_p_* for each type of mechanical test (tension and compression) and each type of material (PETG and ASA). Table 13 presents optimization objectives for each studied material.

For the desirability study, we used the following relationships [3]:

(3) $$D=(d_{1}\cdot d_{2}\cdot \ldots {\;\cdot \;d}_{n})1$$

(4) $$d_{i}=0,\;if\;y_{i\;}<L_{i}\;d_{i}=\frac{\left(y_{i}-L_{i}\right)\cdot r_{i}}{\left(T_{i}-L_{i}\right)},\;if\;L_{i}\leq y_{i\;}\leq T_{i}\;d_{i}=1,\;if\;y_{i\;}>T_{i}$$

where D—composite desirability; *n*—number of responses; $d_{i}—$the desirability for each individual response, $y_{i},\;L_{i},\;T_{i}$—the predicted value, target value, and lowest value, respectively, of the analyzed response of response.

Table 14 shows the composite desirability for each printing parameter and each type of material.

**Figure 7:**
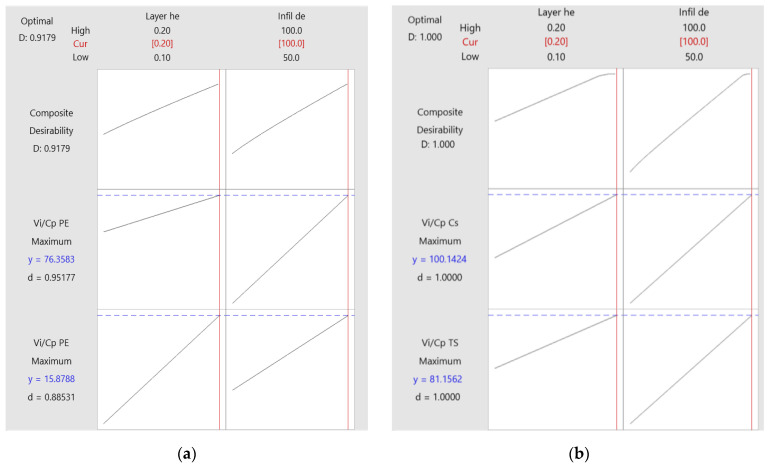
Optimization plots for 3D-printed materials: (**a**) PETG; (**b**) ASA.

Figure 7 shows the plots of FDM parameter optimizations for the manufacture of PETG and ASA samples.

Analyzing Figure 7, we observe how each factor (column) influences the composite desirability response (row). The vertical solid red lines indicate the current setting of the factors, and the red numbers on each column indicate the current level of the factors. The blue horizontal dashed lines indicate the responses corresponding to the current factor settings, and the blue numbers indicate the response corresponding to the current factor settings.

According to Figure 7a, following the optimization process of the FDM parameters for PETG, the results of the optimal settings were as follows: layer height (L_h_) = 0.20 mm and infill density (I_d_) = 100%. Analyzing Figure 7b, we notice that following the optimization process of the FDM parameters for ASA, the results of the optimal settings were as follows: layer height (L_h_) = 0.20 mm and infill density (I_d_) = 100%. Increasing the layer height per pass (L_h_) has a significant impact on print time; this leads to lower power consumption, and thus lower production costs. The decrease in the height of the layer deposited at a pass (L_h_) has a direct impact on production costs, but also on maintenance costs, which increase considerably.

## 4. Conclusions

This paper presents the results of a technical–economical study regarding the optimization of FDM parameters for the manufacture of PETG and ASA parts. In this context, we carried out multi-objective optimization with the aim of finding the optimal FDM parameters (L_h_—the height of the deposited layer in one pass; I_d_—the filling percentage) for the manufacture of PETG and ASA parts. Following the determination of the mechanical characteristics (tensile and compression) of the specimens manufactured via FDM from PETG and ASA, but also the determination of the production cost for each set of specimens, using the fundamental principle of value analysis by maximizing the *V_i_*/*C_p_* ratio, we achieved the technical–economical optimization of the FDM parameters.

Layer height at one pass (L_h_) and infill density (I_d_) are crucial parameters for 3D printing via FDM. This conclusion is highlighted in many studies, such as [4,33,34,35,36,37,48,49,50,51]. L_h_ has an impact on layer adhesion, surface finish, and defects, and a smaller layer height generates higher tensile strength and compressive strength. I_d_ has an impact on the internal structure; a higher infill density generates higher tensile and compressive strength.

The results of the ANOVA show that the two FDM parameters considered (L_h_—the height of the layer deposited in one pass; I_d_—the filling percentage) influence the results of the *V_i_*/*C_p_* ratios. For tensile specimens made of PETG, the parameter that significantly influences the results of the *V_i_*/*C_p_* ratios is L_h_, the height of the layer deposited in one pass, and in the case of compression specimens made of PETG, the parameter that significantly influences the results of the *V_i_*/*C_p_* ratios is I_d_—the filling percentage.

In the case of specimens manufactured via FDM from ASA, the parameter that decisively influences the results of the *V_i_*/*C_p_* ratios of the tensile and compression specimens is I_d_—the filling percentage.

Using the results of the *V_i_*/*C_p_* ratios for the tensile and compression specimens made of PETG and ASA, we found the optimal FDM parameters: L_h_ = 0.20 mm and I_d_ = 100%.

The results of the study have applicability for the efficient exploitation of 3D printers for the manufacture of PETG and ASA parts via FDM.

For the next direction of study, our proposition is to extrapolate the study to other types of materials such as recycled PETG and recycled ASA, but also to other types of mechanical tests such as resilience, flexural, and hardness testing. Also, we want to perform microscopic analyses on parts to investigate microstructure and interface adhesion condition. For the ASA and recycled ASA parts, we plan to choose a broader range of infill densities, I_d_ = (25; 50; 75; 100) %, and layer heights, L_h_ = (0.10; 0.15; 0.20; 0.25; 0.30), mm to capture non-linear trends of ASA parts. For the achievement of the desired objectives, a new FDM 3D printer was purchased (Piocreat G5 Pro), and we were able to manufactured samples via FDM from granular material.

## Figures and Tables

**Table 1:** The main additive manufacturing technologies [31].

Technology Name	Draw	Components	Details
Stereolitograpgy, (SL).	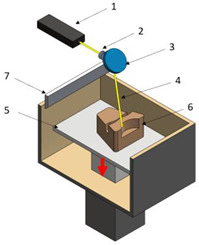	1—laser generator; 2—optic system; 3—galvanometric mirror; 4—laser beam; 5—construction platform; 6—piece; 7—blade.	**Advantages:** + high accuracy of parts; + high print speed. **Disadvantages:** - laborious post-processing of printed parts; - fragility of parts.
Digital exposure of light, (DEL).	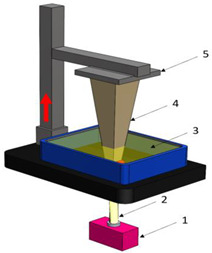	1—digital projector; 2—UV light; 3—resin; 4—piece; 5—construction platform.	**Advantages**: + high quality of surfaces; + high print speed. **Disadvantages:** - high-cost materials; - limited print volume.
Layered manufacturing by laminating layers, (LMLL).	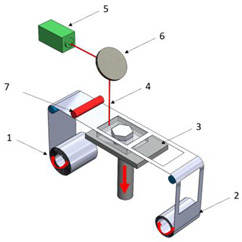	1—driven roller; 2—driving roller; 3—construction platform; 4—laser beam; 5—laser generator; 6—galvanometric mirror; 7—heated roller.	**Advantages**: + high-accuracy parts; + high-stability structures. **Disadvantages**: - significant loss of material. - laborious post-processing of printed objects.
Thermoplastic extrusion, (TE).	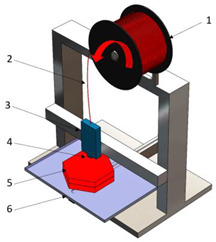	1—coil with material; 2—filament; 3—extruder; 4—extrusion nozzle; 5—piece; 6—construction platform.	**Advantages**: + simple technology; + low-cost materials and equipment. **Disadvantages**: - poor-quality surfaces of parts; - low printing speed.
Selective laser sinterising, (SLS).	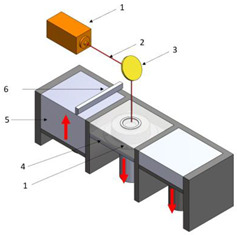	1—laser generator; 2—laser beam; 3—galvanometer 4—construction platform; 5—raw material container; 6—blade.	**Advantages:** + high-resistance parts; + good precision of parts. **Disadvantages:** - poor quality of surfaces poor; - high-cost equipment and materials.
3D inkjet printing (3DP).	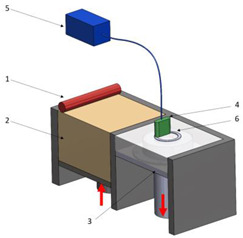	1—scraper blade; 2—enclosure with raw material; 3—work platform; 4—print head; 5—binder tank; 6—track.	**Advantages:** + high printing speed; + reduced costs for materials and equipment. **Disadvantages:** - fragile parts; - poor quality of surfaces poor.
Selective laser melting, (SLM).	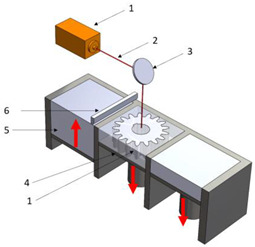	1—laser generator; 2—laser beam; 3—galvanometer 4—construction platform; 5—raw material container; 6—blade.	**Advantages:** + use of high-performance materials; + high resistance of parts. **Disadvantages:** - high-cost equipment and materials; - long duration required for cooling parts.
Polyjet printing with photopolymers, (PJP).	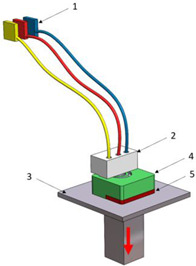	1—liquid polymer tanks; 2—print head; 3—construction platform; 4—piece; 5—piece support.	**Advantages:** + good precision; + simple post-processing operations. **Disadvantages:** - weak resistance of parts; - high-cost materials.

**Table 2:** FDM printing parameters used to manufacture tensile and compressive samples from PETG and ASA [48,49,50,51].

Printing Parameters	PETG	ASA
Part orientation, P_o_	X–Y	X–Y
Extruder temperature, E_t_	250 °C	240 °C
Platform temperature, P_t_	70 °C	90 °C
Printing speed, P_s_	30 mm/s	30 mm/s
Infill pattern, I_p_	Grid	Grid
Layer height, L_h_	0.10/0.15/0.20 mm	0.10/0.15/0.20 mm
Infill density, I_d_	50/75/100%	50/75/100%
Plate adhesion, P_a_	Brim	Brim

**Table 3:** Recommended printing parameters and physical properties of Everfil PETG and ASA filament.

Materials	Recommended Printing Parameters		Physical Properties		
	Extruder Temperature, (°C)	Platform Temperature, (°C)	Density, (g/cm^3^)	Flexural Modulus, (MPa)	Charpy Impact Strength, (kJ/m^2^)
PETG ASA	220–250 240–260	70–90 90–110	1.29 1.07	2200 2100	33 23

**Table 4:** Testing conditions and samples dimensions for experimental investigation.

Mechanical Test	Testing Condition	Sample Dimensions
Tensile	ASTM D638-14 [52] speed 5 mm/min	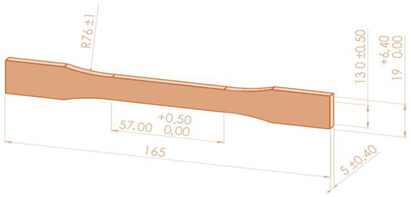
Compression	ISO 604:2002 [53] speed 10 mm/min	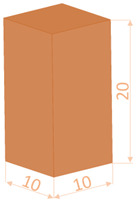

**Table 5:** Cost calculation for PETG samples used for tensile testing.

Sample Set	L_h_, (mm)	I_d_, (%)	$C_{mat}$, (Euro)	$C_{en}$, (Euro)	$C_{p}$, (Euro)
1	0.10	100%	0.99	1.01	2.00
2		75%	0.86	0.69	1.55
3		50%	0.73	0.60	1.33
4	0.15	100%	0.99	0.63	1.63
5		75%	0.86	0.48	1.34
6		50%	0.73	0.43	1.16
7	0.20	100%	0.99	0.51	1.51
8		75%	0.86	0.35	1.22
9		50%	0.73	0.31	1.04

**Table 6:** Cost calculation for ASA samples used for tensile testing.

Sample Set	L_h_, (mm)	I_d_, (%)	$C_{mat}$, (Euro)	$C_{en}$, (Euro)	$C_{p}$, (Euro)
1	0.10	100%	1.04	1.01	2.05
2		75%	0.90	0.69	1.59
3		50%	0.76	0.60	1.36
4	0.15	100%	1.04	0.63	1.67
5		75%	0.90	0.48	1.38
6		50%	0.76	0.43	1.19
7	0.20	100%	1.04	0.51	1.55
8		75%	0.90	0.35	1.26
9		50%	0.76	0.31	1.07

**Table 7:** Determination of *V_i_*/*C_p_* ratio for tensile samples made from PETG.

Sample Set	Ultimate Tensile Strength, (MPa)	$C_{p}$, (Euro)	*V_i_*/*C_p_*
1	28.25	2.00	14.11
2	22.66	1.55	14.64
3	18.76	1.33	14.10
4	25.34	1.63	15.56
5	19.85	1.34	14.82
6	16.61	1.16	14.38
7	24.29	1.51	16.11
8	18.72	1.22	15.40
9	15.48	1.04	14.87

**Table 8:** Determination of *V_i_*/*C_p_* ratio for tensile samples made from ASA.

Sample Set	Ultimate Tensile Strength, (MPa)	$C_{p}$, (Euro)	*V_i_*/*C_p_*
1	43.24	2.05	21.12
2	26.01	1.59	16.39
3	22.69	1.36	16.63
4	40.13	1.67	23.98
5	23.46	1.38	17.01
6	20.87	1.19	17.56
7	39.87	1.55	25.66
8	21.46	1.26	17.09
9	18.82	1.07	17.51

**Table 9:** Cost calculation for PETG samples used for compressive testing.

Sample Set	L_h_, (mm)	I_d_, (%)	$C_{mat}$, (Euro)	$C_{en}$, (Euro)	$C_{p}$, (Euro)
1	0.10	100%	0.22	0.29	0.51
2		75%	0.22	0.19	0.41
3		50%	0.22	0.16	0.38
4	0.15	100%	0.22	0.20	0.42
5		75%	0.22	0.13	0.35
6		50%	0.22	0.11	0.33
7	0.20	100%	0.22	0.15	0.37
8		75%	0.22	0.10	0.32
9		50%	0.22	0.08	0.30

**Table 10:** Cost calculation for ASA samples used for compressive testing.

Sample Set	L_h_, (mm)	I_d_, (%)	$C_{mat}$, (Euro)	$C_{en}$, (Euro)	$C_{p}$, (Euro)
1	0.10	100%	0.23	0.29	0.52
2		75%	0.23	0.19	0.42
3		50%	0.23	0.16	0.39
4	0.15	100%	0.23	0.20	0.43
5		75%	0.23	0.13	0.36
6		50%	0.23	0.11	0.34
7	0.20	100%	0.23	0.15	0.38
8		75%	0.23	0.10	0.33
9		50%	0.23	0.08	0.31

**Table 11:** Determination of *V_i_*/*C_p_* ratio for compressive samples made from PETG.

Sample Set	Compressive Strength, (MPa)	$C_{p}$, (Euro)	*V_i_*/*C_p_*
1	30.33	0.51	59.07
2	19.83	0.41	48.30
3	14.06	0.38	37.19
4	30.57	0.42	72.55
5	20.22	0.35	57.60
6	12.20	0.33	37.03
7	29.20	0.37	78.35
8	19.82	0.32	62.22
9	11.27	0.30	37.28

**Table 12:** Determination of *V_i_*/*C_p_* ratio for compressive samples made from ASA.

Sample Set	Compressive Strength, (MPa)	$C_{p}$, (Euro)	*V_i_*/*C_p_*
1	38.04	0.52	72.65
2	28.58	0.42	67.94
3	16.42	0.39	42.30
4	34.43	0.43	79.78
5	25.85	0.36	71.59
6	15.04	0.34	44.32
7	37.68	0.38	98.45
8	28.54	0.33	86.86
9	16.45	0.31	52.66

**Table 13:** Optimization goals for analyzed materials (PETG and ASA).

Response	Goal	Lower		Target		Weight	Importance
*Vi*/*Cp*		PETG	ASA	PETG	ASA		
Tensile [MPa/EUR]	Maximum	14.10	16.39	16.11	25.66	1	1
Compression [MPa/EUR]		37.03	42.30	78.35	98.45		

**Table 14:** Composite desirability.

Printing Parameters		Material	
Layer Height, (mm)	Infill Density, (%)	PETG	ASA
		Composite Desirability	Composite Desirability
0.10	100	0.453350	0.56643
	75	0.168040	0.29452
	50	0.000000	0.01430
0.15	100	0.696297	0.80768
	75	0.405383	0.44291
	50	0.066163	0.05602
	100	0.917938	1.00000
0.20	75	0.615557	0.59118
	50	0.275221	0.09752

## Data Availability

Data are contained within the article.
